# Can methods of artificial intelligence aid in optimizing patient selection in patients undergoing intrauterine inseminations?

**DOI:** 10.1007/s10815-021-02224-y

**Published:** 2021-05-24

**Authors:** Nejc Kozar, Vilma Kovač, Milan Reljič

**Affiliations:** 1 Department of Reproductive Medicine and Gynaecological Endocrinology, Clinic for Gynaecology and Perinatology, University Medical Centre Maribor, Ljubljanska 5, 2000 Maribor, Slovenia; 2 Faculty of Medicine, University of Maribor, Taborska ulica 8, 2000 Maribor, Slovenia

**Keywords:** Artificial intelligence, Intrauterine insemination, Random forest, Partial least squares, Neural network

## Abstract

**Purpose:**

AI and its machine learning algorithms have proven useful in several fields of medicine, including medically assisted reproduction. The purpose of the study was to construct several predictive models based on clinical data and select the best models to predict IUI procedure outcomes.

**Methods:**

Clinical data (patient baseline characteristics, sperm quality, hormonal status, and cycle data) from 1029 IUI procedures performed in 413 couples stimulated by clomiphene citrate, letrozole, or gonadotropins were used to build several models to predict clinical pregnancy. The models included ANN, random forest, PLS, SVM, and linear models using the caret package in R. The models were evaluated using ROC analysis by means of random CV on test data.

**Results:**

Out of the best performing models, the random forest model achieved an AUC of 0.66, a sensitivity of 0.432, and a specificity of 0.756. This performance was followed by the PLS model, which achieved a sensitivity of 0.459 and specificity of 0.734. The other models achieved significantly lower AUCs. When adjusting the predictive cutoff value, confusion matrices show that clinical pregnancy is twice as likely in the case of positive prediction.

**Conclusion:**

Among the compared methods, the random forest and PLS models demonstrated superior performance in predicting the clinical outcome of IUI. With additional research and clinical validation, AI methods may be successfully used in improving patient selection and consequently lead to better clinical results.

## Introduction

Infertility is a common medical issue, defined as the inability to achieve clinical pregnancy after 1 year of regular, unprotected intercourse. It is estimated to affect 8–12% of reproductive-aged couples worldwide [1]. Although several treatment options exist, only up to 57% of patients seek help [2].

Artificial intrauterine insemination (IUI) is often used as a method of choice for subfertile couples, especially couples with ovulatory dysfunction, unexplained infertility, or mild male factor [3]. Despite lower success rates than in vitro fertilization (IVF), it is still favored by many clinicians because it is more affordable and less invasive than other methods [4]. There is no general consensus on when IUI should be performed. While some experts, e.g., the National Institute for Health and Care Excellence (NICE), oppose the use of IUI for unexplained infertility, studies have shown no difference between 3 IUI cycles and a single IVF cycle. Furthermore, significantly higher live birth rates (LBRs) were found in IUI than in expectant management [5, 6]. If IUI is to be used efficiently, patient selection is critical. Several studies have already investigated prognostic factors with a basic general consensus around the importance of age, duration of infertility, and type of infertility. Many authors have confirmed the importance of sperm quality, exposing a total motile sperm count above 5 × 10^6^ to be suitable for the procedure. Nevertheless, in addition to all known prognostic factors, we still lack methods to optimally select suitable patients and are therefore unable to significantly improve the outcomes [7–9].

In the field of assisted reproductive techniques, artificial intelligence (AI) methods were first employed back in 1997, when artificial neural networks (ANNs) were first used to predict IVF outcomes based on clinical information [10]. Later attempts employed the use of support vector machine (SVM), ANN, and random forest models that included both patient-level clinical characteristics and embryo morphological data. These attempts were far superior to conventional statistical methods in predicting clinical outcomes but were usually of limited clinical value due to lack of external validation [11–14]. The field of AI in medically assisted reproduction has attracted much interest in recent years. At the annual congresses of the two most influential reproductive medicine societies, the American Society for Reproductive Medicine (ASRM) and the European Society for Human Reproduction and Embryology (ESHRE), as many as 18 abstracts on the topic were presented, mostly covering the assessment and embryo selection [15]. However, to the best of our knowledge, no studies are currently investigating the use of AI in IUI.

The aim of this study was to build several machine learning models and select models with the best predictive value to identify couples who may benefit the most from IUI procedures.

## Materials and methods

### Patients

This retrospective observational study included 1029 cycles of IUI performed in 413 couples between 2017 and 2020 at a single tertiary infertility center. Prior to the study, institutional review board (IRB) approval (UKC-MB-KME-44/19) was obtained.

Medical documentation, consisting of medical history, hormonal status, transvaginal ultrasound examination, and sperm analysis, of all the couples was obtained.

Chlamydia trachomatis serology and endoscopic confirmation of tubal patency were performed by either laparoscopy or transvaginal hydrolaparoscopy under local or general anesthesia. The type of procedure was selected at the discretion of the physician. Sperm quality analysis was performed according to strict Kruger criteria.

A set of recorded variables together with patient characteristics and comparisons between pregnant and nonpregnant women is shown in Tables 1 and 2.

**Table 1:** Comparison of the baseline patient characteristics between pregnant and nonpregnant women

Variable	Pregnant (n = 124)	Nonpregnant (n = 905)	p
Age – female (years, IQR)	31.27 (5.0)	31.11 (5.0)	0.26
Age – male (years, IQR)	33.93 (7.25)	34.16 (7.0)	0.70
Duration of infertility (years, IQR)	2.45 (1.5)	2.45 (1.0)	0.47
FSH (IU/L, IQR)	5.66 (1.7)	5.45 (1.9)	0.26
AMH (μg/L, IQR)	5.24 (4.8)	4.79 (3.68)	0.81
BMI (kg/m^2^, IQR)	26.41 (9.0)	24.62 (7.0)	0.57
No. of previous IUI cycles (n, IQR)	0.77 (1.0)	0.95 (1.0)	< 0.01
Primary infertility (n, %)	80 (65)	595 (66)	0.67
Cause of infertility			0.17
Unexplained (n, %)	61 (49)	467 (52)	
Female factor (n, %)	40 (32)	291 (32)	
Male factor (n, %)	7 (6)	84 (9)	
Mixed (n, %)	16 (13)	63 (7)

**Table 2:** Comparison of cycle-specific characteristics between pregnant and nonpregnant women

Variable	Pregnant (n = 124)	Nonpregnant (n =	p
FSH dosage (IU, IQR)	246.0 (381.25)	227.0 (300.0)	0.26
Day of trigger (days, IQR)	14.06 (3.0)	13.27 (4.0)	**0.01**
Max follicle size (mm, IQR)	18.07 (2.85)	17.89 (3.0)	0.98
Avg follicle size (mm, IQR)	18.09 (2.7)	17.94 (3.0)	0.39
No. of follicles > 17 mm (n, IQR)	0.87 (1.0)	0.76 (1.0)	0.24
No. of follicles 14 <> 17 mm (n, IQR)	0.64 (1.0)	0.58 (1.0)	0.32
No. of follicles > 14 mm (n, IQR)	0.78 (1.0)	0.68 (1.0)	0.69
Endometrial thickness (mm, IQR)	8.06 (2.9)	8.40 (3.0)	0.54
Ejaculate volume (mL, IQR)	3.54 (2.1)	3.22 (1.7)	0.31
Sperm concentration (no./mL, IQR)	54.02 (49.1)	45.29 (44.5)	0.28
Motile sperm. concentration (no./mL, IQR)	22.98 (26.7)	17.54 (20.9)	**0.01**
Total sperm count (n, IQR)	16.73 (19.73)	13.21 (15.98)	0.356
Sperm injection volume (mL, IQR)	0.73 (0.2)	0.72 (0.18)	0.316
Stimulation type			**0.04**
Gonadotropins (n, %)	59 (48)	554 (61)	
Clomiphene citrate (n, %)	55 (44	301 (33)	
Letrozole (n, %)	7 (6)	36 (4)	
Natural cycle (n, %)	3 (2)	13 (1)	
Sperm quality grade			**0.04**
Appropriate	94 (76)	590 (65)	
Less appropriate	24 (19)	230 (25)	
Inappropriate	6 (5)	85 (9)	

### Intrauterine insemination

The standard course of treatment for couples with either unexplained infertility, anovulatory infertility, mild endometriosis, or mild male factor consisted of 1–4 cycles of IUI with either gonadotropin stimulation, clomiphene citrate, or, in some cases, even letrozole or natural cycles.

In all the stimulated cycles, clomiphene citrate (N = 356), letrozole (N = 43), or gonadotropins (N = 613) were screened using vaginal ultrasonography before stimulation and followed up during stimulation.

For controlled ovarian hyperstimulation (COH), clomiphene citrate was used at dosages from 50 to 150 mg daily (Clomid, Pantheon France SAS, France) from the 5th to the 9th day of the menstrual cycle. In the case of stimulation with gonadotropins, we began stimulation using recombinant follicle-stimulating hormone (rFSH) (Gonal, Merck Serono, Switzerland) with an initial dose of 37.5 IE daily in the form of subcutaneous injection beginning on the 5th day of the menstrual cycle. In total, 250 mcg of choriogonadotropin alpha (Ovitrelle, Merck Serono, Switzerland) was applied when the size of the follicles increased up to 17 mm in the case of gonadotropin stimulation and up to 20 mm in the case of clomiphene citrate.

There was strict control of the number of follicles. We allowed a maximum of 3 follicles measuring more than 14 mm. Where more follicles were counted, the IUI procedure was not performed, and women were discouraged from having unprotected sexual intercourse in the following days. After a few cycles (N = 16), IUI was performed during their natural cycles.

Sperm samples were collected from the male partners 4 h prior to the IUI procedure following 2–3 days of sexual abstinence. Spermatozoa were concentrated using the swim-up method and evaluated based on the number of progressively motile specimens.

IUI was performed either 24 or 36 h after human chorionic gonadotropin (hCG) injection in the lithotomy position using a Kitazato IUI catheter (Kitazato Medical Co., Ltd., Japan). The women were encouraged to rest in the same position for another 15 min after the procedure was completed.

All the women were instructed to conduct urine pregnancy tests 14 days after the IUI procedure, and those with positive results were invited for examination during which the quantitative level of hCG was measured. Clinical pregnancy was defined as a positive hCG test with an ultrasonically confirmed gestational sac and a confirmed fetal heartbeat.

In all the women, the following data were collected using a standardized data collection form: the age of both partners, duration of infertility, semen parameters, cause of infertility, type of ovarian stimulation, and ultrasonic status prior to hCG injection.

### Patient population

A database was created based on the demographics of both partners, previous treatment, and infertility workup, including the cause of infertility, BMI, and hormonal analysis. Respective IUI cycles were also recorded with respect to the stimulation type and dosage, type of trigger, follicle measurements, and detailed sperm analysis. The exclusion criteria were women > 42 years old and FSH > 15 IU/L, severe endometriosis, severe male factor infertility, and cycles with no or overresponse to ovarian stimulation. Therefore, the population consisted mainly of younger women with expected good ovarian reserve, which is consistent with indications for the IUI procedure. Out of the 1029 IUI cycles, 528 were in couples with unexplained infertility, 331 were in isolated female factor infertility, and 89 were in isolated male factor infertility. In the women’s group, 617 IUI cycles were performed for unexplained infertility, 147 for anovulatory infertility, 53 for unilateral tubal damage, 53 for endometriosis, 63 for a combination of different causes, and 69 for other reasons. In the male partners, 864 IUI cases tested as normozoospermia. All 32 variables were statistically analyzed to determine the individual correlation values. A sample size calculation was performed using pmsampsize by calculating the R-squared value. The calculation estimated 1142 IUI cycles to be the optimal number for desired model building [16].

### Statistical analysis and AI methods

All the analyses were performed using RStudio with R version 4.0.2. For the statistical analysis, base R functions were used with generalized linear models (GLMs) for the continuous variables and Cochran-Mantel-Haenszel tests for the continuous variables. For the machine learning calculations, the caret package version 6.0-86 and caretEnsemble version 2.0.1 were used with appropriate package dependencies. Additionally, the MLeval package was used for model testing, and pmsampsize was used for sample size estimation. Based on our own unpublished results, random forest, PLS, linear and polynomial SVM, ANN, and naïve Bayes models were selected.

### Data preprocessing

Prior to model building, the data were carefully inspected and preprocessed for analysis. Missing values were filled using the down-up principle. Overall, 2.9% of values were recognized as missing. Testing for zero and near-zero variance predictors revealed a single variable, which was removed from the dataset. All the categorical variables underwent one-hot encoding. Scaling and centering were performed on 21 variables, and 20 variables were further processed by Yeo-Johnson power transformation. This type of transformation was selected for its superior results when empirically compared to Box-Cox or exponential transformations. Altogether, 10 variables were left unchanged [17].

### Model training and evaluation

Prior to model building, the data were split into training and testing sets by randomly allocating 70% of cases to the training set. Furthermore, after empirical testing, the following models were selected: SVM models, a PLS model, a GLM, a random forest model, and a multilayer perceptron model. Repeated k-fold cross-validation (CV) was used with k = 10 and 10 repeats. Due to highly imbalanced data, the synthetic minority oversampling technique (SMOTE) algorithm was used to mitigate data inequality. Balancing was performed only on the training set, while the testing set underwent only scaling, centering, and power transformation. Receiver operating characteristic (ROC) curve analysis was selected as the method for calculating the evaluation metrics [18]. An AI system consists of multiple models built upon a unified preprocessed data frame.

Validation of the system was performed by random data allocation into training and test sets and by k-fold CV with 10 iterations. The performance indices for the construction and validation of the models were the area under the ROC curve (AUC), the sensitivity, and the specificity. After individual model assessment, confusion matrices were built and analyzed based on the testing set predictions. Variable importance calculations were performed on applicable models. A subset analysis was performed separately with models built using only baseline characteristics (only the first successive procedures included the data on age, type of infertility, cause of infertility, hormonal status, duration of infertility, and sperm quality). Additionally, the same subset analysis was also performed on respective stimulation protocols with gonadotropins and clomiphene citrate.

## Results

### Statistical analysis

Univariate analyses were performed to test individual variables. Continuous variables were tested by building a GLM for each variable with the patient ID as the blocking variable to adjust for the effect of repeated measures. Categorical variables were tested using the Cochran-Mantel-Haenszel test. The results of the comparison together with the clinical characteristics are shown in Tables 1 and 2. The mean age of the women was 31.2 years old (21–42 years old) and the mean BMI was 25.2 kg/m^2^ (18–53 kg/m^2^). The overall clinical pregnancy rate was 12.1%, with a 10.4% multiple pregnancy rate. The statistical analysis demonstrated that the age of the females, BMI, day of the trigger, number of follicles > 17 mm, sperm concentration, motile spermatozoa concentration, total sperm count, number of successive IUI procedures, and stimulation type were statistically significant (p < 0.05).

### Model evaluation

**Fig. 1:**
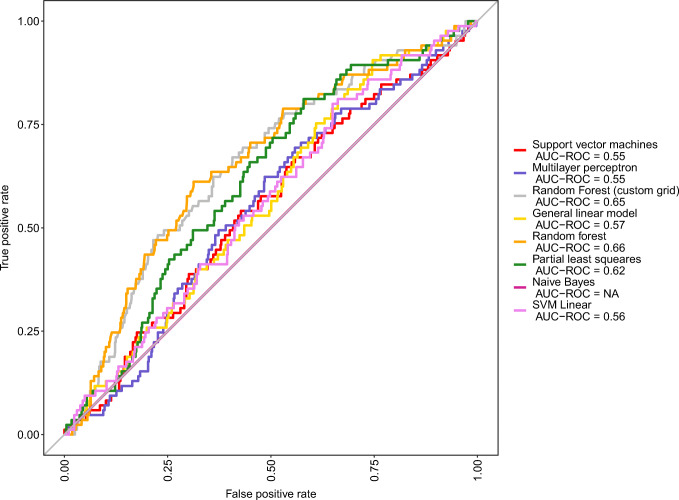
ROC analysis of different ML models

After data preprocessing, 1029 procedures in 413 couples and 37 variables were used to build the respective models using a 0.7 train/test split ratio. Several models were built using the caretList function to provide a comparable set of models. The model set consisted of a GLM, a random forest model, a PLS model, a naïve Bayes model, a linear SVM model, a polynomial SVM model, and a multilayer perceptron model (using three layers with 6, 4, and 2 nodes, which was found to be superior by our empirical trials). There was only a slight difference in the performance of the different models, with the highest accuracy seen in the random forest and PLS models. The best performing model was the random forest model with mtry = 2, built with the randomForest R library, using 36 predictors and 2 classes, a random 0.7 split, and 10-fold CV with 10 iterations. The model achieved an AUC of 0.66. Additionally, a high AUC was also achieved by the PLS model (AUC = 0.62). The PLS model was built with the same split ratio and CV parameters. The best results were achieved with ncomp = 2. The rest of the models, including the ANNs, appeared inferior with respect to the AUC, as shown in Fig. 1.

### Variable evaluation

The univariate analysis revealed the statistical significance of some of the sperm parameters, including the motile sperm concentration and sperm quality grade. Additionally, next to the sperm parameters, the day of the trigger, type of stimulation, and successive IUI procedures were found to be significant with p < 0.05. The models were built with all the variables, irrespective of their statistical significance. When analyzing the variable importance of the two best performing models, the results were generally similar to those of the univariate analysis, where the sperm parameters proved to be most significant. The top 10 variables with their respective importance levels (scaled between 0 and 100) are shown in Table 3.

**Table 3:** Variable importance

Random forest		Partial least squares	
Variable	Importance	Variable	Importance
Total sperm count	100.00	Motile sperm. concentration	100.00
Motile sperm. concentration	84.23	No. of follicles > 17 mm	96.83
Motile sperm. count	78.62	Total sperm count	93.49
Duration of infertility	77.24	Motile sperm. count	84.99
BMI	76.40	Clomiphene dosage	76.02
Max follicle size	72.49	Type of stimulation	74.25
Sperm concentration	71.40	Sperm quality grade	71.11
No. of follicles > 17 mm	69.35	Sperm concentration	67.91
Day of trigger	67.42	Day of trigger	67.28
Ejaculate volume	66.65	Max follicle size	66.56

### Model performance on the test set

The built models were tested on a randomly allocated, imbalanced test set. The default random forest model produced a confusion matrix with 93% correctly predicted negative outcomes and only 5% correctly predicted positive outcomes. As this result is of little use in clinical practice, the cutoff value was adjusted to 0.4, which yielded a model with a lower overall accuracy (0.712), but with a higher clinical value, as shown in Table 4. Similar results were seen in the case of the PLS model. In both models, the positively classified cases were twice as likely to actually achieve clinical pregnancy.

**Table 4:** Confusion matrix of the random forest and PLS models on the test set

	Random forest		Partial least squares	
Pred/obs	Negative	Positive	Negative	Positive
Negative	205	21	219	20
Positive	66	16	72	17
Accuracy	0.712		0.701	
95% CI	0.6637, 0.7671		0.6705, 0.7732	
Sensitivity	0.43243		0.45946	
Specificity	0.75646		0.73432	

### Model performance on different stimulation protocols

A subset analysis was performed on different stimulation protocols to evaluate the importance of separate analyses. The performance of the models differed only slightly between stimulation with gonadotropin or clomiphene citrate. The random forest model achieved an AUC of 0.63 in the gonadotropin group and 0.61 in the clomiphene citrate group. Similar results with AUCs of 0.61 for gonadotropins and 0.59 for clomiphene citrate were achieved by the PLS model.

### Model performance on baseline characteristics

Another attempt was made to build the previously best performing model on a newly prepared dataset, consisting only of the couples’ baseline characteristics. Only the first successive procedures were selected along with age, type of infertility, cause of infertility, duration of infertility, and semen quality. Both the random forest and PLS models achieved a lower performance with AUCs of 0.6.

### Final results

The best model was the random forest model, which achieved an AUC of 0.66, a sensitivity of 0.432, and a specificity of 0.756. It was closely followed by the PLS model, which achieved a sensitivity of 0.459 and a specificity of 0.734. The other models achieved significantly lower AUC values. After optimizing the cutoff value, the confusion matrices showed that clinical pregnancy was twice as likely to occur in the case of positive prediction.

## Discussion

With the rise and accessibility of technology, AI has benefited many aspects of our lives. As it can be applied to medicine, we might witness many improvements in the field of personalized medicine. To what extent AI may affect future infertility treatment can be seen from the number of topics presented at the recent ASRM/ESHRE meetings, where 16 different AI approaches were presented in a single year [15].

As AI applications grow extensively, there are currently no data on the use of AI approaches in the field of IUI. However, several studies have been performed on IVF [19–22].

Although there are no AI studies on IUI procedures, some studies have evaluated the predictive value of different clinical parameters to improve procedure outcomes. Lemmens et al. concentrated on the effect of sperm parameters on IUI outcomes. As expected, a study confirmed a positive relationship between normal sperm morphology and the number of progressively motile spermatozoa, with odds ratios (ORs) of 1.39 and 0.42 for < 1 million motile spermatozoa [23]. In contrast, a meta-analysis by Kohn et al. concluded that sperm morphology had no effect on IUI success rates [24]. In our findings, spermatozoa count and motility were found to be the most important variables. Michau et al. evaluated the effect of the clinical characteristics of both male and female partners. They demonstrated the best LBR in patients with anovulatory infertility compared to other indications, such as endometriosis, unexplained infertility, and unilateral tubal factor [25]. Additionally, female partner age and number of preovulatory follicles had important predictive value in addition to sperm parameters, all confirming the validity of our models. The effect of IUI timing has also been described by Lee et al., where IUI performed more than 36 h after ovulation triggering yielded superior results [26].

Other studies investigating AI application in infertility mostly focused on IVF procedures using ANNs. Apart from clinical data investigations, much focus has been placed on ascertaining efficient embryo selection, where convolutional neural networks have recently played a major role [27]. Important work in the field was performed by Khosravi et al., where Google’s Inception model was used for embryo image analysis in conjunction with decision trees incorporating visual and clinical data. A study showed that AI algorithms outperform embryologists in predicting blastocyst quality and IVF outcomes [28].

Our study shows the potential of different predictive models in predicting IUI procedure outcomes and identifies variables with the highest predictive values. Since AI and machine learning cover a wide array of methods, we believe it is very important to also study other models apart from ANNs since no superior model exists. In our study, we found different results with various models related to their respective strengths and limitations. The AUCs of the best two models (up to 0.66) show only modest performance, which is significantly lower than other studies predicting the outcome of other procedures, such as IVF, with a sensitivity and specificity of 76.7% and 73.4%, respectively [14]. As IUI procedures have overall low success rates that have not significantly improved in recent decades, one cannot expect predictions to mirror those from superior IVF procedures. However, with an optimized cutoff value, a significant positive predictive value may be noted, with positively classified cases achieving clinical pregnancy in 19% of cases. Nevertheless, such high pregnancy rates can hardly be expected in clinical practice due to the very strict selection required for achieving such results, meaning that only a small fraction of couples would actually be selected for the procedure, leaving out a significant number of those who would otherwise also achieve pregnancy. Attempts were made to increase the negative predictive value (NPV) of the models by modifying the cutoff value to identify couples who should avoid IUI treatment. Nevertheless, the NPV could reach as high as 0.91, and further modification led to a great loss of true positive predictions.

Regarding method selection, model performances followed closely. Interestingly, the ANN, employed with different numbers of layers and nodes, proved to be inferior to many other studies where ANN achieved promising results [19–22]. In our case, the random forest algorithm was superior, with PLS following closely. Both random forest and PLS models are known to perform well with strongly intercorrelated data, especially PLS models, which are generally used in chemistry and chemometrics [29]. Multicollinearity in our database was probably one of the reasons for the good performance of those models. On the one hand, random forest models are known to be very efficient in highly complex data and tend to be less prone to overfitting, but they may change significantly due to minor changes in the data. On the other hand, PLS is a statistical method and applies entirely different methodologies. According to the model performances, both models, regardless of the mechanics, perform similarly with both having a very similar sensitivity and specificity and therefore clinical implications. Nevertheless, variable importance selection generally outlines similar variables to other studies. Among the highly intercorrelated variables, the importance may be prone to changes even among iterations of a single model.

A subgroup analysis was performed on the two most frequent stimulation protocols. The built models performed similarly, therefore eliminating potential selection bias. The model could be used regardless of the stimulation type, which could lead to increased robustness and wider clinical application. Nevertheless, the stimulation type was found to be an important predictive factor in the overall models. Additionally, a subgroup analysis was also performed on only the first successive cycles with baseline characteristics, including sperm quality. The models also achieved similar performances, therefore reducing the effect of repeated measures, since no standardized procedures exist for repeated measures in AI.

The main limitation of the study is the selection of variables, since cycle characteristics were included rather than relying only on basal clinical characteristics. Some of these variables are not known prior to the procedure, which is the case with the most important variables, namely, the sperm parameters. Samples are collected and analyzed at the time of insemination. However, sperm are also analyzed prior to the procedure to select suitable patients. The literature states that sperm quality fluctuation is negligible considering clinical significance, although the effect on AI model performance has yet to be tested. On the one hand, analysis of cycle data rather than baseline characteristics may limit the predictive value for initial patient selection, while on the other hand, such models may provide valuable predictions for future procedure outcomes in patients who have already initiated IUI treatment.

Additionally, the study consisted of non-homogeneous group of patients regarding different stimulation protocols, drug usage, timing of procedure after HCG injection, etc. This non-homogeneity was partially addressed by performing several different subgroup analyses. Nevertheless, potential selection bias cannot be completely excluded. Further studies should be performed using strict inclusion criteria and evaluate different groups of patients separately.

Another limitation is the use of multiple predictive models that may render the entire study a bit perplexing though we believe it is of paramount importance to highlight different performances of the models used. The models were built on a uniformly preprocessed dataset which may present another downside. Different models perform better with different preprocessing methods; therefore, targeted and optimized data preparation may lead to improved individual model performance. Further studies are therefore required with individual fine tuning to optimize the best models. Furthermore, a model’s performance has to be tested with well-selected clinical data to ensure robustness and to truly evaluate its effect if used in adjunction to select optimal couples for IUI procedures.

## Conclusions

IUI is a well-known procedure that is still commonly used due to its low cost and relative noninvasiveness. The procedure has not been significantly changed during the years, even with many new predictive factors evaluated. The key to successful IUI procedures is therefore proper patient selection rather than improving the procedure itself. While certain knowledge about the predictive value of clinical data can guide patient selection, AI methods may take a step further, elegantly combining the predictive values of respective variables into one singular prediction. The confusion matrix calculations revealed a twofold difference in the clinical pregnancy rate in the test group based on model prediction. Since patients undergoing IUI are already carefully selected, AI models may be of great assistance in re-evaluating patient selection criteria and classifying borderline cases in particular.
