# Correction to: Can methods of artificial intelligence aid in optimizing patient selection in patients undergoing intrauterine inseminations?

**DOI:** 10.1007/s10815-021-02264-4

**Published:** 2021-06-28

**Authors:** Nejc Kozar, Vilma Kovač, Milan Reljič

**Affiliations:** 1 Department of Reproductive Medicine and Gynaecological Endocrinology, Clinic for Gynaecology and Perinatology, University Medical Centre Maribor, Ljubljanska 5, 2000 Maribor, Slovenia; 2 Faculty of Medicine, University of Maribor, Taborska ulica 8, 2000 Maribor, Slovenia

**Correction to: Journal of Assisted Reproduction and Genetics**

10.1007/s10815-021-02224-y

The article Can methods of artificial intelligence aid in optimizing patient selection in patients undergoing intrauterine inseminations?, written by Nejc Kozar, Vilma Kovač and Milan Reljič, was originally published electronically on the publisher’s internet portal on May 24, 2021 without open access. With the author(s)’ decision to opt for Open Choice the copyright of the article changed on June 17, 2021 to © The Author(s) 2021 and the article is forthwith distributed under a Creative Commons Attribution.

The original article has been corrected.

